# The Role of Histone Deacetylases in Acute Lung Injury—Friend or Foe

**DOI:** 10.3390/ijms24097876

**Published:** 2023-04-26

**Authors:** Guoqing Luo, Bohao Liu, Tinglv Fu, Yi Liu, Boyang Li, Ning Li, Qing Geng

**Affiliations:** Department of Thoracic Surgery, Renmin Hospital of Wuhan University, Wuhan 430060, China; guoqingl@whu.edu.cn (G.L.);

**Keywords:** histone deacetylase, Sirtuin, acute lung injury, macrophage, vascular endothelial cell (VEC), alveolar epithelial cell (AEC), neutrophil, epithelial-mesenchymal transition (EMT), endothelial glycocalyx (EG)

## Abstract

Acute lung injury (ALI), caused by intrapulmonary or extrapulmonary factors such as pneumonia, shock, and sepsis, eventually disrupts the alveolar-capillary barrier, resulting in diffuse pulmonary oedema and microatasis, manifested by refractory hypoxemia, and respiratory distress. Not only is ALI highly lethal, but even if a patient survives, there are also multiple sequelae. Currently, there is no better treatment than supportive care, and we urgently need to find new targets to improve ALI. Histone deacetylases (HDACs) are epigenetically important enzymes that, together with histone acetylases (HATs), regulate the acetylation levels of histones and non-histones. While HDAC inhibitors (HDACis) play a therapeutic role in cancer, inflammatory, and neurodegenerative diseases, there is also a large body of evidence suggesting the potential of HDACs as therapeutic targets in ALI. This review explores the unique mechanisms of HDACs in different cell types of ALI, including macrophages, pulmonary vascular endothelial cells (VECs), alveolar epithelial cells (AECs), and neutrophils.

## 1. Introduction

ALI/ARDS is caused by a variety of factors, such as systemic pathological processes (sepsis, burns, shock, etc.), inhalation (toxic particles or gases, gastric contents, etc.), physical injury (radiation damage, etc.), biological factors (SARS-CoV-2, Streptococcus pneumoniae, Klebsiella pneumoniae, etc.), and therapeutic agents (mechanical ventilation, haemodialysis, etc.). After D. G. Ashbaugh et al. [1] reported acute respiratory distress syndrome (ARDS) in 1967, the AECC meeting in 1994 proposed that 200 mmHg < PaO_2_/FiO_2_ < 300 mmHg should be called ALI after the exclusion of cardiogenic pulmonary oedema, while PaO_2_/FiO_2_ < 200 mmHg should be called ARDS [2], and the Berlin meeting in 2012 replaced ALI with mild and moderate ARDS [3]. ALI and ARDS are different stages of the same disease, with ALI being earlier and less severe. The underlying aetiology of ALI/ARDS leads to the disruption of the alveolar-capillary barrier, and increased permeability of the former due to inflammatory mediators, with protein-rich fluid leaking from the vessels into the alveoli, causing diffuse pulmonary oedema. In addition, alveolar macrophages and recruited monocytes express a large number of pattern recognition receptors to recognize pathogen-associated and risk-associated molecular patterns, thereby initiating a signaling cascade that produces and secretes a large number of cytokines and chemokines, recruits neutrophils and other immune cells, and also promotes cell death by various mechanisms that further amplify the overall inflammation in ALI/ARDS. Consistent with the aforementioned mechanisms, ALI/ARDS presents with diffuse imaging changes in the lungs bilaterally, as well as restricted pulmonary gas exchange, i.e., intractable/refractory hypoxemia, as in the Berlin definition where PaO_2_/FiO_2_ is negatively correlated with severity [4].

ALI/ARDS has a high morbidity and mortality rate—10% of patients diagnosed with ARDS in intensive care units (ICUs) in 50 countries and a 46.1% mortality rate in patients with severe ARDS in 2016, and this syndrome seems to be underappreciated [5]. There are also many sequelae in surviving ALI/ARDS patients, such as pulmonary dysfunction and cognitive impairment [6]. In the 55 years from 1967 to the present, aside from symptomatic treatment measures such as positive end-expiratory pressure ventilation (PEEP), prone ventilation, and nutritional support, which have some effect, no specific treatment has been found to address the pathophysiological mechanisms of ALI/ARDS, and treatment measures are too limited [7,8]. Therefore, the search for therapeutic approaches that can target the molecular mechanisms of ALI/ARDS has become particularly important.

To address this need, numerous clinical trials have been conducted to find effective therapies. New therapeutic tools: (1) mesenchymal stem cells (MSCs). The ability of MSCs to multidirectionally differentiate and self-renew, along with the privilege to evade immunity, makes it possible to rescue and regenerate damaged cells in ALI/ARDS. Only ALI is described below. In a 2015 clinical trial, the feasibility of MSCs for the treatment of ALI was confirmed, and no serious adverse effects were observed [9]. In addition, the use of MSC extracellular vesicles (EVs) for the treatment of ALI is a novel attempt and awaits further study [10]. Recently, researchers have also used synthetic iron oxide-based nanoparticles (SPIONs) labeled/pretreated MSCs (SPION-MSCs) to treat ALI, which not only enhanced the viability of MSCs, but also promoted the conversion of macrophages to the M2 state [11]. (2) PamHRchol/GA mixed micelles. Cholesterol was conjugated to histidine- and arginine-grafted polyamidoamine (PamHR) for micelle formation. The cholesterol-conjugated PamHR (PamHRchol) was mixed with amphiphilic GA to produce PamHRchol/GA mixed micelles. It was confirmed that PamHRchol/GA micelles could reduce LPS-associated ALI inflammation, and in addition, micelles gene delivery efficiency was high, suggesting the possibility of micelles delivering anti-inflammatory genes to ameliorate ALI [12]. (3) Fibrinolytic. Inhibition of fibrinolytic activity in bronchoalveolar lavage fluid (BALF) is one of the characteristics of ALI. An animal study showed enhanced fibrinolysis in BALF in the experimental group of fibrinolytic agents compared to the control group, and more importantly, improved lung function and inflammatory response in an experimental animal model of ALI [13]. This suggests the possibility of fibrinolytic treatment of ALI. These studies are novel explorations with preliminary results. In the search for a treatment for ALI, epigenetics-based studies are gradually increasing.

Post translational modifications (PTMs) cause “minor” variations in proteins, and they include acetylation [14], phosphorylation [15], methylation [16], ubiquitination [17], ADP-ribosylation [18], and citrullination [19]. However, these supposedly insignificant variations can have a significant influence on how proteins work. These PTMs are synthesized much more quickly than new proteins, and the combination of different types with different numbers of PTM composition causes each of the same proteins to exhibit distinct activities and actions, to adapt to various environmental and signaling stimuli. Growing evidence suggests that these PTMs are strongly linked to lung inflammatory diseases. For example, the K433 residue of mitochondrial pyruvate kinase M2 (PKM2) deacetylation alleviates lung ischemia/reperfusion (I/R) injury [14]; the ability of protein phosphorylation to regulate a variety of cellular processes is well known, as the AMPK phosphorylation can improve ALI [15]; the METTL3 (Methyltransferase 3)-induced N6-methyladenosine (m6A) of GPX4 can regulate sepsis-induced ALI [16]; TRAF2 (TNF receptor associated factor 2) ubiquitination aggravates lipopolysaccharide (LPS)-induced ALI [17]; NFATc3 ADP-Ribosylation modulate macrophage inflammatory gene expression in LPS-induced ALI [18]; protein citrullination is a PTM occurring in the pulmonary district (and adjacent area), resulting in an alteration of several functional effects of these cells. Recent research has shown that protein citrullination increased in diesel exhaust particle-induced-normal human bronchial epithelial (NHBE) cells, which suggests a positive correlation between protein citrullination and cell inflammation [19], although citrullination is currently less well studied, its association with inflammation has brought it increasing attention.

The first and best-studied PTM, acetylation, is controlled by both histone/lysine acetylases (HATs/KATs), the writers [20], and histone/lysine deacetylases (HDACs/KDACs), the erasers, at lysine (K) residues at the amino terminus of histones and non-histone proteins. Deacetylases with HDAC in their titles, such as HDAC1, and HDAC10, are referred to as members of the classical HDAC family. Deacetylases with Sirt in their titles, such as Sirt1, and Sirt7, are referred to as members of the Sir2 family, also known as Sirtuin. It should be noted that although both of these families are discussed in this review and are mammalian HDACs, HDACs refers to both the classic HDAC family and the Sir2 family, whereas HDAC inhibitor (HDACi) only refers to inhibitors against the classic HDAC family. If there is any special information, it will be explained.

HDACs are named because they were first discovered in the process of histone deacetylation, an enzyme that removes acetyl groups from histone lysines, and because the “writing” of acetyl groups usually correlates positively with transcriptional activation. In contrast, HDACs usually inhibit transcription; therefore HDACs are also known as transcriptional co-repressors [21,22]. There is growing evidence that most HDACs play an important role in non-histone proteins such as microtubule proteins, cell cycle proteins, and signaling proteins, regulating different processes and reactions that occur in cells [23,24]. Moreover, a few HDACs can play a role in removing long-chain acyl groups, in addition to acetyl groups [25]. Thus, the role of HDACs goes far beyond their nomenclature. When reflecting on the place of HDACs in ALI, one must consider the wide range of substrates they act on to gain insight into their specific role.

Abnormal acetylation of proteins usually leads to diseases, and regulation of their writers and erasers is of great importance. PTMs can cause qualitative changes for even small fine changes in histones, so HDACs, as the enzyme that erases acetyl groups, must be regulated with precision. Its enzymatic activity is also regulated by phosphorylation, SUMOylation (ubiquitin-like protein modifying molecule), etc. [26].

Since the discovery of the first mammalian HDAC called HDAC1 in the United States in 1996 [27], more than 12,000 studies with the keyword “HDAC” have been published in Pubmed, indicating that HDACs are indeed a focal point worthy of further investigation. The approval of HDACis in clinical use [28], prompts consideration of the possibilities of HDACs in the treatment of ALI.

## 2. 18 HDACs

To date, a total of 18 HDACs have been identified in mammals. These 18 mammalian HDACs are homologous to different *Saccharomyces cerevisiae* (also known as “Baker’s Yeast” or “Brewer’s Yeast”) protein sequences, act on different substrates, and localize to different intracellular compartments (Table 1). They are classified according to the mechanism of action into the Zinc-dependent classical HDAC family (also known as the histone deacetylase family) and the NAD+ (nicotinamide adenine dinucleotide)-dependent silent information regulator 2 (Sir2) family (also known as the Sirtuin). Additionally, the 18 HDACs were divided into four categories based on homology, with *S. cerevisiae* proteins and a structural similarity: class I reduced potassium-dependent 3 (RPD3)-like protein (HDAC1, HDAC2, HDAC3, HDAC8); class II histone deacetylase 1 (HDA1)-like protein (HDAC4, HDAC5, HDAC6, HDAC7, HDAC9, HDAC10); class II HDACs are divided into class IIa (HDAC4, HDAC5, HDAC7, HDAC9) and class IIb HDACs (HDAC6, HDAC10); class III Sir2-like protein (Sirt1, Sirt2, Sirt3, Sirt4, Sirt5, Sirt6, Sirt7); and class IV (HDAC11) [29,30]. It should be added that yeast HDA one similarity 1 (HOS1), HOS2, and HOS3 proteins also have 35–49% homology with RPD3p (yeast protein)—so class I HDACs are also homologous to HOSp.

The classical HDAC family includes class I, II, and IV HDACs, all of which are Zinc -dependent enzymes that are dependent on the presence of zinc ions in exerting their catalytic activity. The Sir2 family includes the remaining class III HDACs, which, unlike the classical HDAC family, exert deacetylation with NAD^+^ as a cofactor, and are NAD^+^-dependent enzymes that deacetylate and transfer the acetyl group to the ribose C2 position of NAD^+^, ultimately producing the deacetylation products, 2′-O-acetyl-ADP ribose, and nicotinamide [31]. The removal of acetyl groups is a reversible reaction, and nicotinamide is a potent inhibitor of Sirtuins, as it acts as a product of the deacetylation reaction. The artificial determination of these families, as well as classes, is not as absolute as one might think, and there may be a functional and structural crossover between different families or classes of HDACs, which must be recognized when probing their acting substrates.

All HDACs are named according to the order of their discovery; for example, in April 1996, HDAC1 was isolated and purified from an affinity substrate by a deacetylation inhibitor, trapoxin, with sequence similarity to RPD3p [27]; in November 1996, Yang et al. identified another HDAC2 [32] with sequence homology to RPD3p; in 1997, HDAC3 was discovered with 53% sequence similarity to HDAC1 [33], and so on. The Sir2 family is homologous to the brewer’s yeast Sir2p, named independently of the classical HDAC family. Sir2 family promotes silent heterochromatin formation in yeast [34], and was found to be conserved in bacteria to humans in 1995 [35], the Sirtuin family in humans and mammals was subsequently discovered and named chronologically.

### 2.1. Classical HDAC Family

#### 2.1.1. Class I HDACs

Class I HDACs include HDAC1, HDAC2, HDAC3, and HDAC8. Class I HDACs are similar to the RPD3p, which promotes yeast histone deacetylation and ultimately leads to gene silencing [36]. Class I HDACs are generally considered to be expressed unrestrictedly among species, within species, and are primarily localized to the nucleus.

In 1996, Taunton et al. isolated and purified an RPD3-like protein from mammals named histone deacetylase 1 (HDAC1) using an HDACi, trapoxin [27]. What was not known at the time when the enzyme was given its name was that, like other HDACs discovered later, their acting substrates were not limited to histones and acetyl groups. Later in the same year, while researching the mechanism of gene regulation by a mammalian zinc finger transcription factor, YY1, Yang et al. identified another RPD3-like protein, HDAC2, which is used by YY1 as a cofactor to implement gene silencing [32]. After identifying two HDACs homologous to the RPD3p, the researchers further searched the Genbank database for HDACs with similar protein and DNA sequences to HDAC1/HDAC2 and found HDAC3, which has 50–55% similarity to HDAC1/HDAC2 in both DNA and protein sequences, and is also an RPD3-like protein [33]. Similarly, in 2000, HDAC8 was discovered [37].

HDAC1 and HDAC2 are localized in the nucleus and, because of their proximity to histones, these two HDACs are known as true histone deacetylases, as they are the mainstay of histone acetylation removal. HDAC1/HDAC2 exist in the nucleus as homo- and heterodimers in the NuRD (nucleosome remodeling deacetylase), Sin3A (septation initiation network transcriptional regulatory protein 3), CoREST (corepressor RE1 silencing transcription factor), and MiDAC (Mitotic deacetylase complex) transcriptional repressor complexes, whose activity suffers a reduction when detached from the complex. They share more than 80% sequence similarity, which is why we often discuss the two HDACs together [28]. HDAC3 is competitively regulated by nuclear export signals (NES) and nuclear localization signals (NLS) and can shuttle between the nucleus and cytoplasm [38]. Unlike HDAC1/HDAC2, HDAC3 usually forms a complex with the NCoR (nuclear receptor corepressor)/SMRT (silencing mediator of retinoid and thyroid receptors) and has enhanced deacetylase activity upon binding, as confirmed by numerous studies [39,40]. Unusually, HDAC8 is the only HDAC isoform with a gene located on the X chromosome, localized in the nucleus and cytoplasm, and does not bind any complexes but exerts deacetylation alone [41]. Unlike other class I HDACs, the activity of HDAC8 is reflected in the hydrolysis of long-chain acyl lysines, and Lin et al. demonstrated that HDAC8 hydrolyzed longer acyl chains of octanoyl-, dodecanoyl-, and myristoyl-lysines with higher activity than acetyl lysine [25].

It is commonly accepted that class I HDACs are widely expressed and localized in the nucleus, but studies to date have confirmed that there are some notable exceptions—HDAC3 is restrictedly expressed between tissues, and HDAC3 and HDAC8 can shuttle between the nucleus and cytoplasm.

#### 2.1.2. Class II HDACs

Class II HDACs include class IIa (HDAC4, HDAC5, HDAC7, HDAC9) and class IIb (HDAC6, HDAC10), which are separated into class IIb due to the presence of two deacetylase structural domains in HDAC6 and HDAC10. Class II HDACs are homologous to HDA1p, which is homologous to and has a similar function as RPD3p, the active subunit of HADp, and has the same role in removing acetyl groups to promote gene silencing in yeast [36]. Class II HDACs are localized in the nucleus and cytoplasm and are restricted in expression between tissues.

After the discovery of class I HDACs similar to RPD3p, since HADp is structurally and functionally similar to RPD3p, researchers tried to search the Genbank database for *human* HDACs with similar sequences to HDA1p and discovered three class II members—HDAC4, HDAC5, HDAC6—in 1999 [42]. SMRT can act on nuclear hormone receptors to assist in gene silencing, and HDAC7 was discovered while exploring its transcriptional mechanism of action. HDAC7 shares 46% and 42% amino acid sequence identity with HDAC4 and HDAC5, respectively [43]. Shortly after the discovery of HDAC7, HDAC-related protein (HDRP) [44], and MEF-2 interacting transcriptional repressors [45,46] were identified. These proteins were subsequently confirmed to be a new member, the alternatively sheared isoform of HDAC9 [47]. Similarity to HDAC6 led to the discovery, naming, and classification of a protein—HDAC10 [48].

Class IIa HDACs include HDAC4, HDAC5, HDAC7, and HDAC9, and all of these enzymes can be found in the nucleus and cytoplasm, suggesting that, in addition to their ability to act on histones, class IIa HDACs are also closely associated with the regulation of non-histones in the cytoplasm. Class IIa HDACs are specially expressed in the tissue, such as HDAC4 in the brain, heart, and skeletal muscle tissues, HDAC5 in the brain, heart, skeletal muscle, and placental tissues, HDAC7 in the heart and lung, and HDAC9 in the brain and skeletal muscle more than other tissues. The much lower in vitro deacetylation activity of class IIa HDACs compared to class I HDACs is due to the replacement of conserved tyrosine by histidine in the structural domain of deacetylases in vertebrates, as demonstrated by an interesting phenomenon—the HDAC4 His-976-Tyr mutation is 1000-fold more efficient than WT, which can be extended to HDAC5 and HDAC7, indicating the necessity of conserved tyrosine residues for maintaining deacetylase activity and the evolutionary changes in the structure.

#### 2.1.3. Class IV HDACs

Class IV HDACs include only one member, HDAC11, which was discovered in 2002, and is mainly localized in the nucleus, it is highly expressed in the kidney, heart, brain, skeletal muscle, and testis [49]. The study confirmed that the activity of HDAC11 is reflected in the hydrolysis of long-chain acyl lysines, such as dodecanoyl, myristoyl, and ε-N-myristoyl lysines [50,51]. Its catalytic activity is inhibited by the products of the deacylation reaction, such as free myristic acid, palmitic acid, and stearic acid, while its activity is promoted by acyl-providing substrates such as palmitoyl CoA and myristoyl CoA.

### 2.2. Sir2 Family

#### Class III HDACs

Class III HDACs include Sirt1, Sirt2, Sirt3, Sirt4, Sirt5, Sirt6, Sirt7. Brachmann et al., in 1995, proposed that the Sir2 family is conserved from bacteria to humans, and that they are closely associated with histone deacetylation [35]. Subsequently, an attempt was made in the Genbank database to find human Sir2 homologs with similar amino acid sequences to the yeast Sir2p, and five Sirtuins were found—Sirt1, Sirt2, Sirt3, Sirt4, and Sirt5, with Sirt1 being the most similar to the Sir2p sequence [52]. Similarly, the remaining two members-Sirt6 and Sirt7 were found using the amino acid sequence of Sirt4 as a probe [53]. The Sir2 family is grouped into HDACs due to its histone deacetylation function and exists as special HDACs.

Mammalian Sirtuin members exhibit different subcellular localization and substrate specificity, and are involved in different biological processes such as metabolism, inflammation, oxidative stress, and aging. The Sir2 family is expressed unrestrictedly in cells, Sirt1 and Sirt2 are localized in the nucleus and cytoplasm, Sirt3 is localized in the nucleus and mitochondria, Sirt4 and Sirt5 are found only in the mitochondria, Sirt6 is only in the nucleus, and Sirt7 is present in the nucleus [54]. The Sir2 family removes the acetyl group from the acetylated lysine substrate and transfers the acetyl group to the ADP-ribose group of NAD^+^ to generate nicotinamide, 2′-O-acetyl-ADP-ribose, and deacetylation products [55]. In addition to classical histone deacetylation, it has been shown to have ADP-ribosyltransferase activity [56].

## 3. Physiological Functions of HDACs

Acetylation occurs at the ε-amino group of lysine, and HDACs and HATs are responsible for the removal and binding of the acetyl group. Lysine acetylation in histones has long been identified, and new evidence suggests that lysine acetylation exists not only in histones, but also in non-histones [23,24,57].

The common term HDACs generally refers to Zinc-dependent HDACs, the classical HDAC family, which includes the class I, II, and IV HDACs that are essential for the deacetylation of histones and non-histones. Since tyrosine is replaced by histidine in the highly conserved deacetylation structural domain of class IIa HDACs, other classical HDAC family members—except for class IIa—retain tyrosine in their catalytic domains and, thus, have strong deacetylation activity. Histone deacetylation inhibits gene transcriptional activity and transcriptional rate, while non-histone proteins are involved in more cellular processes, such as signaling, cell cycle, cell migration, and inflammatory activation, due to their wide variety [23]. When we understand the substrates of HDAC action, we need to take into account histones and non-histones, which are of equal importance. HDACis play an active role in the treatment and improvement of cancer; inflammatory lung diseases (including asthma, COPD, and lung injury); cardiovascular disease; diabetes; and fibrosis [28,58,59,60,61], which, in turn, suggests that members of the classical HDAC family play a facilitating role in the progression of these diseases.

The *human* Sir2 family is a highly conserved NAD^+^-dependent deacetylase that ranges from bacteria to humans, and includes class III HDACs named Sirtuins [35,62]. Unlike the classical HDAC family, Sirtuins have a prolonging effect on a biological lifespan. Studies have confirmed that Sirt6 overexpression in mice prolongs lifespan, and that Sirt1 attenuates neurodegenerative diseases by extending cell lifespan [63,64]. *Human* Sirtuins are present in various compartments of the cell, which allows this family to coordinate biological responses throughout the cell [65]. For example, Sirt1, Sirt6, and Sirt7 are localized in the nucleus, and their main function is to deacetylate histones to regulate transcription. While Sirt2, as the only Sirtuin located mainly in the cytoplasm, is responsible for the deacetylation of some cell cycle-related non-histone proteins, signaling non-histone proteins, and assists in mitotic checkpoint function [66,67]. Sirt3, Sirt4, and Sirt5 are specific members of Sirtuins that are localized in mitochondria, and are closely associated with the regulation of mitochondrial homeostasis, mitochondrial oxidative stress, and mitochondrial autophagy [68,69,70]. When we talk about the efficiency of the action of Sirtuins, it is not only the expression of Sirtuins that is considered, but more importantly their enzymatic activity. Sirtuins are NAD^+^-dependent deacetylases, and NAD^+^ is an important intermediate in mitochondrial energy metabolism, so Sirtuins enzyme activity is positively correlated with the NAD^+^/NADH ratio [71], which also makes them sensors of cellular energy status. Sirtuins have also emerged as new targets for the treatment of a variety of diseases, such as cancer, diabetes, inflammatory diseases, and neurodegenerative diseases.

### 3.1. HDACs Regulate Transcription

Nucleosomes are formed by wrapping 146bp of DNA around a histone octamer (H2A*2, H2B*2, H3*2, H4*2) for 1.75 turns, and are the basic structural unit of chromatin. The portion of histone extending outward from the nucleosome is called the histone tail. A variety of PTMs, such as histone acetylation, methylation, ubiquitination, phosphorylation, and SUMOylation, occur on this, and these modifications do not change the gene structure, but can affect the phenotypic changes of the organism, and are called epigenetic phenomena, which can pass on different types and different amounts of histone PTMs in combination to the offspring.

Acetylation modifications occur on the lysine ε-amino group at the amino-terminal tail of histones, and also on non-histones. Histone acetylation modifications have been shown to affect gene activity and transcription rate [72]. The possible mechanisms of action of acetylation modifications related to transcription are as follows: (1) after the acetylation modification of histones, the positive charge of histones is neutralized, which in turn weakens the binding to negatively charged DNA, and the chromatin structure is changed from tight to loose, exposing DNA binding sites and giving a transcriptionally open state to some genes. (2) The bromodomain can read acetylated lysines, so acetylated histones are also able to recruit a variety of molecular chaperones containing the bromodomain to indirectly control transcriptional activity [73,74]. (3) Acetylation of non-histone proteins, such as transcriptional co-activators, transcription factors, and nuclear receptors, which indirectly regulate the transcriptional rate and activity of genes. HDACs exert the opposite effect of the above, compacting chromatin and removing acetyl groups that attract molecular chaperones, while regulating the activity of non-histone proteins such as transcription factors, HDACs usually act to repress transcription.

Evidence linking HDACs to transcriptional repression is the finding that RPD3p is required for full activation and full repression of transcription of multiple target genes [75]. Over time, multiple mammalian HDACs homologous to RPD3p were identified, which also implies that they are likely to play a similar role to RPD3p, and studies proved that this is indeed the case. GAL4 DNA binding domain-mammalian RPD3 (HDAC2) fusion protein strongly represses transcription from promoters containing GAL4 binding sites [32]. Several subsequent similar studies also confirmed that the fusion protein of GAL4 bound to multiple HDACs severely reduced the transcription of DNA possessing GAL4 binding sites [33,76]. This suggests that HDACs are recruited under specific conditions and do not actively function, which puts the gene regulation mechanism in order. In addition, acetylation weakens the binding of histones to DNA, as confirmed by the fact that high acetylation levels of histones reduce the number of linkage changes per nucleosome core particle and that acetylation significantly reduces the binding constant of the H4 “tail” to DNA [77,78,79]. When histones become loosely bound to DNA, it is expected that gene transcription will be enhanced, at least more than that of genes tightly bound to histones, and that HDACs will abolish this change.

Acetylated non-histone proteins are also associated with transcriptional regulation and HDACs are responsible for their deacetylation. The transcription factor p53 plays an immune guardian role and plays an important function in apoptosis and autophagy, which are essential for the clearance of tumor cells. Acetylation of transcription factor p53 can enhance the transcriptional activity of p21 by recruiting it to the p21 promoter via the bromodomain of the TFIID subunit TAF1 [80]. In addition, it was shown that transcription factor IIB (TFIIB) was transcriptionally enhanced after acetylation at lysine 238, compared to the unacetylated TFIIB mutant [81]. Conversely, HDACs that mediate the deacetylation of p53 and TFIIB can reduce transcriptional efficiency. The signal transducer and activator of transcription 6 (STAT6) is a non-receptor tyrosine kinase. It was shown that IL-4 enhances 15-lipoxygenase-1 (15-LOX-1) expression in mammals by enhancing the acetylase activity of CREB-binding protein (CBP)/p300 and promoting the acetylation of STAT6, whereas a histone acetylation inhibitor, sodium butyrate, causes a decrease in 15-LOX-1 expression by blocking the acetylation of STAT6 [82]. Classical HDAC family-mediated non-histone deacetylation usually has a negative effect on transcription by repression. In addition, some members of Sirtuins also exhibit regulatory transcriptional roles. For example, Sirt6 promotes anti-apoptotic gene expression by deacetylating the transcription factor GATA4, which plays a role in improving myocardial apoptosis [83]. Similarly, Sirt7 modulates the transcriptional activity of GATA4 by deacetylating GATA4 and ameliorates phenylephrine-induced cardiac hypertrophy [84]. Sirtuins are undoubtedly also involved in the regulation of transcription by non-histones, and, unlike the classical HDAC family that plays a disease-promoting role, most, though not all, Sirtuins exert their protective effects on the organism through different mechanisms, in line with what has been described here.

It is commonly assumed that genes bind HATs when they are required for transcription and that HDACs replace HATs when they are silenced. The fact that HDACs deacetylate histones and thus compact chromatin is undeniable, but the idea that HDACs need to be combined only in the presence of gene silencing is too absolute. A genome-wide mapping study showed that after CHIP (chromatin immunoprecipitation)-seq experiments, HDACs were found to bind to a variety of transcriptionally active genes as HATs and positively correlated with mRNA transcription of some genes [85]. This may be because transcriptionally active genes undergo acetylation by HATs and subsequently require deacetylation by HDACs to reset chromatin. Additionally, this study shows that HATs and HDACs bind genes usually in a transient response, and histones may undergo multiple acetylation modifications in a short period, while HDACs play a role in maintaining the original state of genes through rapid deacetylation, dynamically regulating the acetylation level of genes with HATs. Overall, this shows that HDACs do not bind to genes when they need to be silenced, due to the extremely short duration of the reversible action of acetylation and deacetylation, which makes HDACs act like a soldier on standby, ready to play a role in removing acetyl groups added by HATs all the time. This is also somewhat different from what we often think of as inhibition of transcription; HDACs are also associated with transcriptional enhancement.

HDACs, as transcriptional co-repressors, can indirectly regulate transcription or even activate transcription, in addition to their direct transcriptional repression function. As described above, HDACs can indirectly enhance transcription by deacetylating transcriptional repressor genes or by deacetylating non-histone proteins that can inhibit transcription [29]. In conclusion, it is possible that HDACs can play both transcriptional repressive and transcriptional activating roles, and the specific indirect or direct regulation and its mechanisms need to be further explored.

### 3.2. HDACs Regulate Non-Histone Substrates

Over the past two decades, numerous proteomic analyses have confirmed that a large number of non-histone proteins are acetylated, and that HDACs mediate their deacetylation [86,87]. These acetylated non-histone proteins are involved in a large number of cellular activities, such as DNA damage repair, cell division, signaling, autophagy, and transcription [23]. Acetylated non-histones and acetylated histones have equal importance, and the non-histones are more numerous and involved in more cellular processes. At the protein level, changes in non-histone function can be achieved by altering the structure of non-histone proteins after conferring non-histone acetyl groups, or by regulating non-histone stability, as well as by altering the localization of non-histone proteins in cellular compartments. On the cellular level as a whole, the combination of different non-histone acetylations coordinates different processes throughout the cell.

The p53 tumor suppressor has been extensively studied as the first identified non-histone target of HDACs and HATs. It is closely associated with apoptosis and autophagy, and an increasing number of studies have highlighted the role of p53 in tumor progression. Previous research has shown that HDAC1-containing complexes can mediate the deacetylation of p53 and inhibit the cell cycle, and the apoptosis-regulating function of p53 [88]. Similarly, Sirt1 protects by removing acetylation of p53 to inhibit p53-dependent apoptosis in response to oxidative stress and to reduce apoptosis sensitivity [89]. The p21 protein inhibits cyclin-dependent kinase (CDK) complex activity and regulates the cell cycle. Acetylated p53 enhances the transcriptional activity of p21, and transcriptionally translated p21 acts synergistically with p53 in the G1 phase checkpoint to intercept unrepaired DNA and reduce cancer cell development [80]. In addition, acetylation of K98, K117, K161, and K162 sites of p53 is quite important for its mediated iron death and oncogenic effects, and loss of acetylation of these four sites exhibited a severe defect in tumor growth inhibition and loss of regulation of the metabolic target SLC7A11 [90]. The removal of p53 by HDACs-mediated acetylation can, on the one hand, attenuate the ability of p53 to induce apoptosis leading to cancer cell development and, on the other hand, reduce the sensitivity of normal cells under stress.

Microtubulin, another widely studied non-histone protein regulated by acetylation, plays an important role in maintaining cell morphology and regulating intracellular transport, mitosis, and cell migration [91]. The K40 site of α-microtubulin is the only acetylation site known to occur on polymeric microtubules, and K40 acetylation can stabilize the structure of α-microtubulin and avoid unstable breakage of intracellular microtubules. Alpha-microtubulin acetyltransferase 1 (ATAT1) catalyzes the acetylation of α-microtubulin at K40 in various organisms from Tetrahymena to humans [92], while HDAC6 is responsible for the removal [93]. Microtubule protein acetylation, a marker of microtubule stability, decreases microtubule stability and, thus, cell morphology instability after HDAC6 removal, while this enhances cell motility migration. In addition, it was shown that microtubule protein acetylation deficiency decreases in vitro molecular motor kinesin-1 binding and transport, while increased microtubule protein acetylation mediated by inhibition of HDAC leads to recruitment of kinesin and kinesin-1 into microtubules, thereby compensating for the intracellular transport defect in Huntington’s disease [94]. HDAC6 is the HDAC that has been shown to play an important role in microtubule deacetylation, and this discovery provides a new target for disease treatment.

In addition, phosphatase (PTEN etc.) [95], nuclease (NBS1 etc.) [96], kinase (CDK9, BTK, p38MAPK etc.) [97,98,99], transcription factor/coactivator/corepressor (PLAG1, ZNF76, FOXO3, RUNX3) [100,101,102,103], are also regulated by HDACs. The fact that HDACs can regulate non-histone proteins is a certainty, since lysine acetylation is truly present in non-histone proteins, and these studies offer a new way of thinking with regard to understanding the function of HDACs.

### 3.3. HDACs Indirectly Regulate Post-Translational Modifications

In addition to acetylation, various PTMs (e.g., methylation, ubiquitination, SUMOylation, and phosphorylation) can occur in the lysine ε-amino group, and there are either direct competitive binding relationships or indirect regulatory relationships between acetylation and these modifications. When the lysine ε-amino acid undergoes acetylation modification, the acetyl group occupies a spatially favorable position and undoubtedly hinders the introduction of other post-translational modifications. Conversely, the binding of other PTMs may be favored after HDACs mediate the erasure of acetyl groups. The earliest evidence found was the competitive binding of ubiquitination and acetylation. The occurrence of lysine acetylation inhibits the introduction of ubiquitination and suppresses proteasome-mediated protein degradation [104]. This suggests that HDACs may promote protein degradation. In addition to competitive binding relationships, the formation of long ubiquitin chains is regulated by acetylation [105]. This suggests a complex interrelationship between acetylation and ubiquitination.

Another important example of crosstalk between lysine acetylation and other PTMs is methylation. Histone lysine methylation is a key player in the regulation of gene expression, cell cycle, genomic stability, and nuclear structure, and acetylation has been shown to crosstalk with methylation. Dot1 (telomere silencing disruptor-1) is a histone H3 lysine 79 (H3K79) methyltransferase that is dysregulated in leukemia. H4K16 is a key chromatin modification site, and in vitro studies have shown that its acetylation (H4K16ac) directly enhances the sevenfold activity of Dot1 and stimulates yeast Dot1 to increase H3K79 methylation in a manner distinct from, but coordinated with, histone H2B ubiquitination (H2BUb) [106,107]. In addition, H3K9ac not only inhibits methylation of the same residue, but also promotes H3K4 methylation, thereby allowing transcriptional activation. HDACs-mediated removal of H3K9ac inhibits H3K4 methylation and ultimately transcriptional activation.

In addition, protein phosphorylation and dephosphorylation mediated by protein kinases and protein phosphatases can inhibit or activate proteins, depending on the amino acid site of action. Dual specificity phosphatases (DUSP) are a class of protein phosphatases that dephosphorylate both phosphoserine/phosphothreonine (pSer/pThr) and phosphotyrosine (pTyr). In diabetic patients, HDAC3 is activated and inhibits *Dusp5* expression by deacetylating histone H3 on the primer region of the *Dusp5* gene, which in turn leads to ERK1/2 activation and the development of diabetic cardiomyopathy (DCM) [108]. Inhibition of HDAC3 function with the inhibitor was followed by ChIP assays showing enhanced acetylation of histone H3 at the *Dusp5* gene promoter, increasing transcription and leading to ERK1/2 inhibition and improved DCM.

The crosstalk between ubiquitination, methylation, phosphorylation, and acetylation shows the breadth of acetylation’s action, not just with regard to the immediate chromatin-opening role of acetylation, which ripples farther than is thought, but also, the role of HDACs far beyond what is thought of as regulating transcription and regulating non-histone roles. The connection between acetylation and the various PTMs is like a vast biological network, and perhaps new combinations of PTMs are occurring where we are not looking. This needs to be thought of when considering side effects when using HDAC inhibitors for treatment.

## 4. Advances in the Studies of HDACs in ALI

HDACs play an important role in cancer, diabetes, neurodegenerative diseases, and inflammation [61,109,110,111]; the classical HDAC family usually plays a disease-promoting role and Sirtuins play a bioprotective function. The role of HDACs in the development of ALI as a new perspective that contributes to the understanding of ALI is unclear.

The pathogenesis of ALI involves multiple cells, including damage to pulmonary vascular endothelial cells (PVECs), activation of macrophages, activation of neutrophil migration leading to the production of various inflammatory factors, and epithelial-mesenchymal transition (EMT) in alveolar epithelial cells(AECs) stimulated by sustained inflammation [112]. HDACs exert deacetylation in histones, as well as in a variety of non-histone proteins, a target worthy of in-depth discussion, and we describe the close association of HDACs with ALI in different cell types.

### 4.1. Macrophages

**Figure 1:**
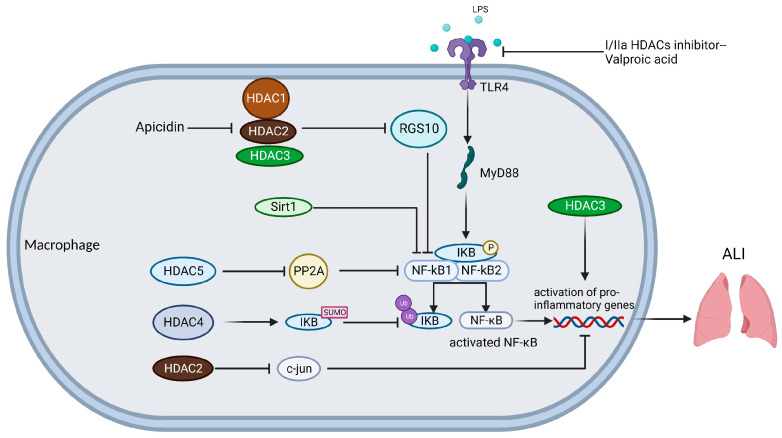
HDACs in macrophages in ALI. In macrophages, LPS activates inflammatory gene expression through the TLR4-MyD88-NF-κB pathway. RGS10, a GTPase-activating protein, can block inflammatory pathway activation. RGS10 in macrophages is inhibited by HDAC1, 2, and 3 in LPS-induced ALI, and this inhibition is rescued by apicidin, thereby ameliorating ALI. c-Jun transcription factor in macrophages binds to inflammatory gene promoters to form a nuclear receptor co-repressor complex, while HDAC2 downregulates c-Jun in LPS-induced ALI. HDAC4 blocks NF-κB pathway activation by promoting SUMOylation of the IKB protein that stabilizes NF-κB to prevent its Ubiquitinated degradation. Macrophage PP2A phosphatase inhibits IKB phosphorylation and stabilizes NF-κB to reduce its nuclear translocation, whereas HDAC5 blocks this effect and promotes exacerbation of LPS-associated ALI inflammation. HDAC3 is also able to directly promote macrophage inflammatory gene expression to promote ALI. In addition, Sirt1 belongs to the Sirtuin family, which can directly inhibit macrophage NF-κB activation. However, more detailed mechanisms of action, such as the action of HDACs on protein deacetylation sites remain to be investigated. These findings suggest that most of the classical HDAC family exerts pro-inflammatory effects in LPS-induced ALI macrophages except for HDAC4, while Sirtuin exerts anti-inflammatory effects. Created by Biorender.com, accessed on 10 Feb 2023.

The classical activation pathway of macrophages makes them pro-inflammatory M1 macrophages in ALI, while the alternative activation pathway makes them anti-inflammatory and pro-fibrotic M2 macrophages [113]. Macrophages express multiple pattern recognition receptors (PRR), which activate downstream signaling pathways by recognizing pathogen-associated molecular patterns (PAMP) and damage-associated molecular patterns (DAMP), while secreting different cytokines to interact with other immune cells [114]. Alveolar macrophages (AM) are specialized cells found in the alveoli and are important innate immune cells of the respiratory system that function as a barrier through phagocytosis [115,116] (Figure 1).

NAD^+^-dependent deacetylases Sirtuins, also known as “longevity genes”, play an effective role in suppressing the production and progression of ALI inflammation mediated by alveolar macrophages. The degree of inflammation and oxidative stress is positively correlated with the progression of ALI. MicroRNAs (miRNAs) are a class of non-coding single-stranded RNAs encoded by endogenous genes that can target mRNAs, inhibit their translation, and destabilize them, thereby down-regulating the expression of target genes, miRNAs play an important role in oxidative stress and cell death [117]. There is growing evidence that miRNAs promote inflammation in ALI by downregulating Sirt1 expression. The downregulation of miR-217 and miR-199a rebounded macrophage Sirt1 levels, and miRNA levels were negatively correlated with Sirt1 levels to achieve anti-inflammatory and antioxidant effects on sepsis-associated ALI in vivo and in vitro, Sirt1 inhibition eliminated the protective effect of miR-217 and miR-199a downregulation on ALI [118,119]. In addition, miR-30d-5p and miR-146a-3p could bind to *Sirt1* mRNA 3’-UTR, decrease *Sirt1* expression level, and further promote NF-κB inflammatory signaling pathway [120,121]. Endoplasmic reticulum stress (ERS), a protective response that restores proteostasis by activating the unfolded protein response (UPR), is an important pathophysiological mechanism of sepsis-associated ALI, and C/EBP homologous protein (CHOP) plays an important role in ERS-induced apoptosis [122,123]. Activation of Sirt1 inhibits ERS in LPS-treated mouse macrophages by reducing CHOP protein while reducing apoptosis of macrophages [124]. In addition, Sirt1 has been shown to mediate the deacetylation of the p65 subunit of NF-κB thereby inhibiting the NF-κB signaling pathway and attenuating the inflammatory response [125]. Some herbal extracts such as aloe-emodin, rhodiol glycosides, curcumin, and a general anesthetic agent propofol can increase the expression level of Sirt1, thus inhibiting the NF-κB signaling pathway and the production of NLRP3 inflammatory vesicles, inhibiting different types of cell death such as apoptosis, pyroptosis and necroptosis, and acting as an ameliorator of ALI [126,127,128].

Sirt3, localized in mitochondria, has been shown to attenuate myocardial toxicity by maintaining oxoguanine-DNA glycosylase-1 (OGG1) levels and protecting mitochondrial DNA (mtDNA) [129]. The development of ALI is strongly associated with mitochondrial dysfunction and the production of reactive oxygen species (ROS). Recent results show that macrophages obtained from Sirt3-specific knockout mice exhibit significant alterations in mitochondrial bioenergetics and redox homeostasis, and that viniferin, a Sirt3 activator, improves mitochondrial homeostasis in LPS-induced macrophages, reduces ROS production and thus attenuates ALI [130]. The autophagy of AMs interacts with their phagocytosis, where autophagy regulates macrophage inflammation and homeostasis by recycling cellular components and ATP [131]. Sirt6 enhances the autophagy of AMs and induces the conversion of M1 to M2 macrophages to reduce macrophage inflammation in ALI [132].

Class I HDACs include HDAC1, 2, 3, and 8, and their presence is essential for macrophage development, homeostasis, and activation. When LPS activates macrophages to generate an in vitro ALI model, class I HDACs usually exert a pro-inflammatory effect and have an ameliorative effect on ALI when knocked down. c-Jun inhibits the transcription of pro-inflammatory genes in macrophages by binding to the promoters of pro-inflammatory genes and forming a nuclear receptor co-repressor complex. It was shown that HDAC2-specific knockdown increases the transcription of the *c-Jun* gene promoter by promoting its acetylation, thereby decreasing the expression of inflammatory factors such as IL-12, iNOS, and TNF-α in LPS-induced macrophages [133]. Another member, HDAC3, regulates mitochondrial function through binding to the peroxisome proliferator-activated receptor γ (PPARG-γ) enhancer, which in turn regulates the development and homeostasis of AMs, and the presence of HDAC3 is also closely associated with the classical and alternative activation pathways of macrophages [134,135,136]. Mohammed et al. showed that HDAC3-specific inhibition decreased LPS-induced inflammatory cytokine secretion in macrophages and also increased the tolerance of macrophages to LPS stimulation [137]. Macrophages release inflammatory factors in response to LPS stimulation, and the ability to release inflammatory factors reflects their immune capacity, and HDAC3 knockdown prevents this response. The regulator of G protein signaling (RGS) acts as a GTPase activating protein, activating GTPase activity in the Gα subunit of G protein, and when GTP changes to GDP, G protein returns to its initial quiescent state, blocking cascade signaling downstream [138]. It has been shown that *RGS10* expression was repressed in LPS-induced AMs and that apicidin, a selective inhibitor of HDAC1-3, blocked this gene silencing and restored the inhibition of *RGS10* expression level on the downstream signaling pathway of G proteins, thereby improving sepsis-associated ALI [139]. Class I HDACs may play a role in inhibiting the action of RGS and promoting the activation of signaling pathways. In addition, class I HDACs could act synergistically or independently with the anti-inflammatory factor IL-4 to regulate LPS-induced secretion of inflammatory mediators from macrophages [140]. This suggests that class I HDACs do not act independently and that they have a broad range of actions with other molecular chaperones.

Class IIa HDACs include HDAC4, 5, 7, and 9, with HDAC4 being the most abundantly expressed in macrophages. Class IIa HDACs are closely associated with the NF-κB pathway in LPS-activated macrophages, and are also associated with inflammatory glycolysis. When cells are exposed to activators such as LPS, TNF-α, and IL-1β, the IκB subunit, which binds to NF-κB dimers and promotes their stability, is phosphorylated, ubiquitinated, and ultimately degraded by the 26S proteasome [141]. The study confirmed that HDAC4 belongs to the SUMO E3 ligase family, which can competitively inhibit ubiquitin binding through SUMOylation IκBα, reduce IκB degradation, maintain NF-κB stability, and reduce NF-κB pathway activation [142]. In addition, salt-inducible kinase 2-mediated HDAC4 phosphorylation in LPS-activated macrophages was inhibited by Escitalopram, a drug used to treat depression, and the HDAC4 phosphorylation level was reduced and shifted to the nucleus, promoting p65 deacetylation and cytoplasmic shuttling and attenuating NF-κB inflammatory pathway activation, thereby improving sepsis-associated ALI [143]. HDAC4, similar to Sirt1, can promote the deacetylation of p65, thereby inhibiting the NF-κB inflammatory pathway, and is one of the few classical HDAC families that can inhibit macrophage inflammation. It was shown that HDAC5 overexpression enhances Mycoplasma pneumoniae (MP)-induced macrophage inflammation through activation of the NF-κB pathway [144]. The phosphatase PP2A can inhibit the transcriptional activation of NF-κB by decreasing the phosphorylation of IKKβ and IκBα. In a similar study, HDAC5 promoted K136 deacetylation of PP2Ac, leading to PP2A inactivation and increased activation of the NF-κB inflammatory pathway, thereby promoting inflammation in LPS-activated macrophages [145]. Pyruvate kinase M isoform 2 (Pkm2) is a glycolytic enzyme that is closely associated with cancer proliferation, energy metabolism, and migration [146]. The study confirms that PKM2 forms a complex with pro-inflammatory HDAC7 in mouse macrophages, that HDAC7 mediates the K433 site deacetylation of PKM2 to license its pro-inflammatory function, and that LPS-induced inflammatory glycolysis is enhanced in mice when HDAC7 is overexpressed [147]. Class IIa HDACs are key intermediates in linking TLR-induced glycolysis to inflammation via PKM2. HDAC9 of class IIa HDACs can also affect alternative activation pathways in macrophages [148].

The association of HDAC6 with LPS-activated macrophages in class IIb HDAC is widely studied. In sepsis-associated ALI, nitric oxide (NO) production is an important factor contributing to vascular injury and cytotoxicity. It has been shown that HDAC6 can activate STAT1 by promoting phosphorylation at Y701 of STAT1, which further increases interferon-regulatory factor-1 (*IRF-1*) expression, leading to increased iNOS levels in LPS-activated macrophages, thus promoting sepsis-associated ALI [149,150]. In addition, HDAC6 overexpression in macrophages dose-dependently induced MAPK activation and also promoted activation of NF-κB and AP-1 signaling pathways, contributing to sepsis-associated ALI [151]. Another similar study showed that specific knockdown of HDAC6 inhibited the LPS-induced TLR4-MAPK/NF-κB signaling pathway in macrophages and promoted activation of the Nrf2-HO-1 anti-inflammatory signaling pathway and improved sepsis-associated ALI [152]. HDAC6 in macrophages is associated with the activation of MAPK, NF-ΚB, and AP-1 inflammatory pathways. miR-22 specifically inhibits HDAC6 expression by binding to the 3’-UTR of HDAC6 mRNA, thereby improving inflammation in LPS-activated macrophages [153]. A significant body of evidence has confirmed the fact that HDAC6 is closely associated with various inflammatory diseases and plays a pro-inflammatory role, and HDACis, which specifically inhibits HDAC6, may become a regulatory agent to improve ALI.

Different HDACis regulate different signaling pathways, some of which can regulate cell death in macrophages. Valproic acid is a class I/IIa HDAC selective inhibitor [154], a biocompatible valproic acid-coupled nanoparticle constructed to inhibit LPS activation of the TLR4-MyD88-NF-κB signaling axis in human macrophages and reduce TNF-α secretion, thereby reducing macrophage inflammation [155]. Samanta et al. tried new epigenetic therapies in their exploration of LPS-induced ALI treatment. They combined a DNA methylation transferase inhibitor (DNMTi) called 5-Aza 2-deoxycytidine (Aza) and HDACi called Trichostatin A (TSA) for ALI, and found that the combination treatment significantly reduced apoptosis due to MAPK pathway activation in LPS-activated bone marrow-derived macrophages (BMDM) compared to untreated patients or those given a non-combination treatment [156]. JMJD3, a JmjC family histone demethylase, exerts pro-inflammatory effects in addition to methylation [157]. The 5-Aza and TSA combination also down-regulated the activation of the STAT3-JMJD3 pathway, probably because the combination of 5-Aza and TSA led to the acetylation of the STAT3 promoter and, thus, the conversion of BMDM to an M2 state; a more detailed mechanism remains to be investigated [156]. In addition, Aza and TSA can reverse LPS-activated macrophage apoptosis and scorch death by restoring the integrity of the mitochondrial membrane [156]. The inhibitory effect of another short-chain amino acid butyrate on HDAC has also been demonstrated long ago [158]; butyrate ameliorates the inflammatory response caused by Staphylococcus aureus by inhibiting HDAC, NF-κB, and IFN-β/STAT1, thereby reducing NO production in macrophages [159]. These HDACis are among the highly promising drugs that may see approval for ALI treatment in the future.

### 4.2. Vascular Endothelial Cells (VECs)

**Figure 2:**
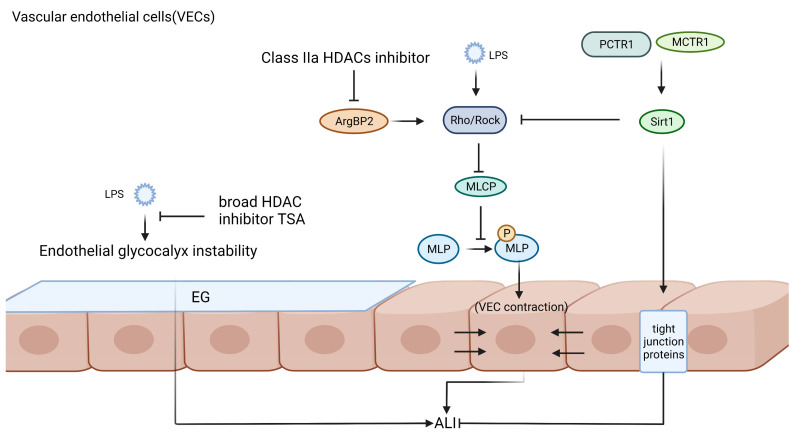
HDACs in vascular endothelial cells (VECs) in ALI. In pulmonary vascular endothelial cells (PVECs), LPS inhibits the phosphorylation of myosin light chain (MLC) phosphatase (MLCP) via the Rho/ROCK pathway, thereby reducing its activity, which increases MLC phosphorylation and drives the onset of contraction in VECs, which leads to the formation of pulmonary edema from protein fluid entering the alveoli. Arginine kinase-binding protein 2 (ArgBP2) in VECs promotes activation of the Rho/ROCK pathway, while the class IIa inhibitor blocks this activation, relieves the contraction of VECs, and attenuates LPS-associated ALI. Sirt1 activation is mediated by inhibition of the Rho/ROCK pathway and the promotion of tight junction protein (TJ) expression to reduce vascular permeability, while Protectin conjugates in tissue regeneration 1 (PCTR1) and Maresin conjugates in tissue regeneration 1 (MCTR1) (novel macrophage-derived lipid mediators) promote Sirt1 activation in VECs and improve LPS-induced ALI. In addition, LPS damages endothelial glycocalyx (EG) attached to the surface of VECs, which not only increases the direct contact between VECs and LPS, but also removes antioxidant enzymes such as SOD3 bound by EG. The broad HDAC inhibitor TSA prevents the damage of EG on the surface of VECs by LPS. Created by Biorender.com, accessed on 10 Feb 2023.

The integrity of the tight junctions between vascular endothelial cells is an important condition to avoid intravascular protein outflow, and one of the important pathogenesis of ALI is the disruption of the integrity of the tight junctions of the pulmonary microvascular endothelium, resulting in the impairment of the air-blood barrier and the transfer of proteins from the blood vessels to the interstitium and alveoli, which eventually leads to alveolar edema and the formation of hyaline membranes [160]. Rho belongs to the Ras superfamily of low-molecular-weight GTPases, also known as Rho GTPases, which are involved in many physiological activities, including cell migration, adhesion, proliferation, and differentiation. Rho-associated kinase (ROCK) is a major downstream target of Rho, a widely expressed serine-threonine protein kinase [161]. LPS activates the Rho/ROCK signaling pathway, thereby targeting myosin phosphatase target subunit 1 (MYPT1) phosphorylation at the T853 site inhibiting myosin light chain (MLC) phosphatase (MLCP), leading to increased levels of MLC phosphorylation and the onset of VECs contraction, disrupting the integrity of the VECs cytoskeleton and tight intercellular junctions, and ultimately leading to a broken VECs barrier and increased permeability [162,163,164] (Figure 2).

Arginine kinase-binding protein 2 (ArgBP2) is an articulatory protein that plays an important role in the regulation of cytoskeletal structure. Overexpression of ArgBP2 promotes the overactivation of RhoA/ROCK signaling and inhibits VECs myosin- and actin-mediated cytoskeletal rearrangements, which are required to maintain vascular lumen integrity [165]. Recent studies have shown that both class IIa HDACs inhibitors and selective HDAC6 inhibitors attenuate the activation of the ArgBP2/Rho signaling pathway in LPS-activated human lung microvascular endothelial cells (HLMVECs), thereby reducing the level of myosin light chain phosphorylation in VECs and restoring cytoskeletal reorganization, reducing the disruption of the VECs barrier in sepsis-associated ALI, and thereby reducing protein fluid leakage [166].

Sirtuins play a protective role in VECs in ALI models. Sirt1 activation increases LPS-induced expression of tight junction proteins (TJs) in VECs, enhancing endothelial junctions to ensure vascular integrity, while this study suggests that Rho/ROCK inhibition may be a target for Sirt1 to reduce endothelial tight junction permeability to mitigate sepsis-associated ALI [167]. In addition, Sirt3 attenuates hyperoxia-induced ALI by increasing manganese superoxide dismutase (MnSOD) production to reduce the level of ROS and control mitochondrial functional stability [168]. The transcription factor nuclear factor red lineage 2-related factor 2 (NRF2) is considered to be one of the major coordinators of the cellular antioxidant response [169]. Sirt6 attenuates LPS-induced ALI by increasing the level of Nrf2 protein and activating anti-inflammatory as well as antioxidant enzyme genes [170]. The silencing of Sirt7 in ECs attenuates LPS-induced expression of inflammatory factors and endothelial adhesion molecules and induces EMT, and the loss of endothelial adhesion molecules is accompanied by an increase in endothelial barrier permeability, which in turn reduces endothelial barrier permeability to mitigate sepsis-associated ALI [171]. Under physiological conditions, VE-calmodulin and β-linked protein exist as a complex, and when inflammation occurs leading to endothelial damage, the VE-calmodulin/β-linked protein complex dissociates and β-linked protein translocates from the cytoplasm to the nucleus, which can promote the expression of Matrix metalloproteinases (MMP)-7 [172,173]. Sirt3 enhances the interaction between VE-calmodulin and β-linked protein by promoting VE-calmodulin expression to stabilize the complex and maintain the integrity of microvascular endothelial adhesion junctions while attenuating inflammation in ECs to reduce LPS-induced ALI [174]. Sirtuins mainly regulate VECs-impaired-mediated vascular permeability and avoid pulmonary edema and hyaline membrane production. 

The loss of endothelial glycocalyx (EG) during the progression of ALI is an important cause of increased pulmonary vascular permeability. EG grows on and within the luminal wall of blood vessels, the most luminal layer of the vessel [175]. Evidence confirms that degradation of EG is associated with substantial disease progression, including sepsis [175,176]. EG is important for maintaining normal endothelial function, while it also binds extracellular superoxide dismutase 3 (SOD3) and protects the endothelium from oxidative damage, and inflammatory mechanisms during sepsis degrade pulmonary EG [177,178]. Recent studies have shown that the broad HDAC inhibitor TSA prevents the degradation of EG and loss of SOD3, reduces the inflammatory permeability of human adipose microvascular endothelium (HAME), and preserves its resistance to oxidative stress [178]. Syndecan-4 (SDC4) is an important proteoglycan of EG, which is a target of MMP-9-targeted degradation shedding in TNF-α-activated endothelial cells, and SDC4 is decreased on the surface of ECs and increased in the culture medium [179]. The deficiency of ECs-specific Sirt1 mediates the shedding of SDC4 outer structural domains by increasing superoxide levels and inducing NF-κB signaling activation, which ultimately impairs EG, and this allows for increased vascular permeability and further amplification of inflammation [180]. Conversely, the presence of ECs Sirt1 stabilizes the intracellular NF-κB pathway, and also stabilizes EG on the cell surface. Protectin conjugates in tissue regeneration 1 (PCTR1) and Maresin conjugates in tissue regeneration 1 (MCTR1) are novel macrophage-derived lipid mediators, and PCRT1 and MCTR1 can downregulate acetyl heparinase (HPA) expression to inhibit LPS-induced EG degradation. However, this effect was abrogated by ALX inhibitors or Sirt1 inhibitors, suggesting that PCRT1 and MCTR1 can restore EG loss in lung ECs via the ALX/SIRT1/NF-κB axis, thereby improving sepsis-associated ALI [181,182].

### 4.3. Alveolar Epithelial Cells (AECs)

**Figure 3:**
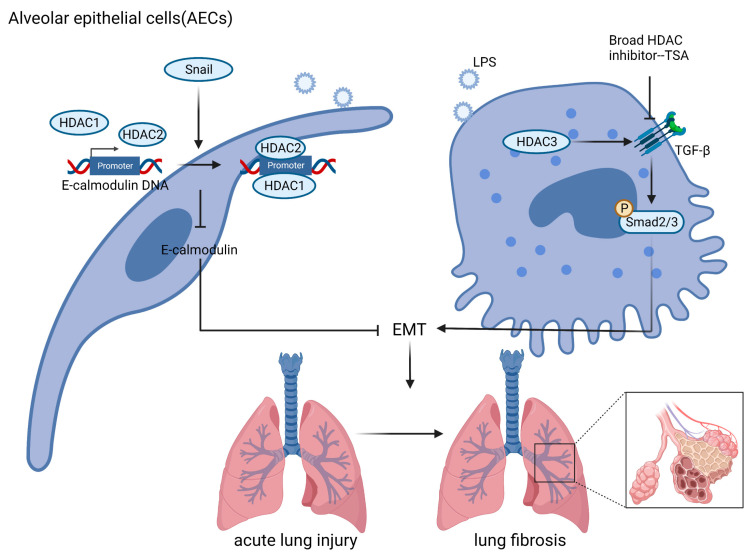
HDACs in alveolar epithelial cells (AECs) in ALI. The transcription factor snail plays an important role in the epithelial-mesenchymal transition (EMT) process in pulmonary fibrosis. The snail can promote the EMT process by recruiting and binding HDAC1 and 2 to the E-calmodulin gene promoter region, where the removal of HDAC1 and 2 from acetylation exerts a transcriptional repressive effect and downregulates E-calmodulin expression. The TGF-β-smad2/3 pathway promotes the EMT process and exacerbates fibrosis. Broad HDAC inhibitor TSA inhibits this process in LPS-stimulated AECs, while HDAC3 plays a facilitating role. Created by Biorender.com, accessed on 10 Feb 2023.

Diffuse damage to AECs is closely associated with the progression of ALI. Intrapulmonary or extrapulmonary factors attack and destroy AECs, causing the release of AECs DAMP, which, in turn, activates macrophages and neutrophils, amplifying the inflammatory response and exacerbating ALI. In addition, AECs are associated with alveolar-capillary barrier permeability, which, as innate immune cells themselves, can express multiple PRRs recognizing different molecular patterns, and upon activation secrete multiple immune factors such as antimicrobial peptides (AMP) and chemokines, which exert antimicrobial and pro-inflammatory effects [183]. EMT is a process in which epithelial cells lose their intercellular junctions and polarity in response to external stimuli or pathological conditions, and then take on the characteristic morphology of mesenchymal cells [184], also is closely related to the transformation of lung injury to pulmonary fibrosis [185,186] (Figure 3).

The transcription factor snail has been shown to promote the EMT process in pulmonary fibrosis [187]. Snail has been described as a direct repressor of E-calmodulin expression during development and carcinogenesis. Peinado et al. [188] found that Snail interacts with HDAC1 and HDAC2, and that Snail can recruit them to bind to the E-calmodulin promoter, forming a repressor complex that deacetylates histones H3 and H4, inhibiting the transcriptional initiation of E-calmodulin and ultimately facilitating the EMT process. Although Snail transcription is also repressed by HDAC1, the phosphorylation of p68 RNA helicase at Y593 dissociates HDAC1 from the Snail promoter, and Snail levels are then upregulated and E-calmodulin expression is blocked, leading to EMT [189]. Similarly, E-cadherin is inhibited by the combined action of snail/HDAC in cancer [190,191]. Recent studies have shown that PM2.5-induced specific knockdown of HDAC3 in AECs inhibits the TLR4-IkBa-NF-κB pathway and TGF-β-Smad2/3 pathway, reduces inflammation production and EMT, and improves PM2.5-associated ALI [192]. This suggests that HDAC3 may mediate EMT following damage to AECs in ALI, and may be a target for the inhibition of pulmonary fibrosis. The broad HDAC inhibitor TSA significantly decreased the level of TGF-β1 protein in radiation-induced type II AECs, inhibited their EMT important signaling pathways, and attenuated their EMT-like morphological changes by decreasing the expression of α-SMA, Snail, and E-calmodulin to attenuate the development of EMT-induced pulmonary fibrosis in AECs in radiation-associated ALI [193]. 

EMT occurs in AECs when the lung suffers, for example, sepsis, and ischemia-reperfusion injury, leading to the mesenchymal formation and irreversible lung fibrosis. There are few studies on alveolar epithelial cells and HDACs in ALI, here, we still found the promoting effect of some HDACs such as HDAC1, HDAC2, and HDAC3 on EMT in ALI and the inhibiting effect of HDACis on EMT in ALI, which can eventually play a protective role against lung injury transformed into lung fibrosis.

### 4.4. Neutrophils

Unlike chronic inflammation, ALI is shorter in duration, and has a greater release of cytokines [194]. The concentration of neutrophils in bronchoalveolar lavage fluid (BALF), a major member of secretory cytokines, was significantly correlated with the severity of ALI [195]. When inflammation occurs, activated macrophages and lung epithelial cells release chemokines, and neutrophils move toward the site of inflammation and release a variety of cytokines, including proteases, reactive oxygen species, pro-inflammatory cytokines, and pro-coagulant molecules [196]. This process also forms neutrophil extracellular traps (NETs), which exert a unique bacterial trapping effect [197]. Although necessary for anti-inflammatory purposes, excessive recruitment and activation of neutrophils can lead to lung tissue damage and further loss of lung function [198].

HDACs regulate neutrophil migration, phagocytosis, the release of pro-inflammatory factors, and the formation of NETs. NETs are reticulated DNA structures produced by neutrophil death with attached granules and nucleoproteins. Although NETs are effective in intercepting and killing pathogens, their formation is also capable of producing local irritation and sterile inflammation. Recent studies have shown that Class I/IIb HDACs can drive the NETs formation from neutrophils and that their inhibitors can reduce local and systemic inflammation [199]. Class IV HDAC-HDAC11 deficiency leads to increased release of pro-inflammatory cytokines by neutrophils and displays enhanced migration and phagocytosis, which can enhance LPS-associated sepsis injury [200]. This, in turn, suggests that HDAC11 attenuates neutrophil migration and activation and reduces the release of inflammatory factors, making HDAC11 one of the members that improve inflammation. Neutrophils secrete leukotriene B4 (LTB4) in response to the formyl peptide N-formylmethionylleucyl phenylalanine (fMLP) [201], a neutrophil chemotactic agent, for migration and chemotaxis. The HDAC inhibitor suberoylanilide hydroxamic acid (SAHA), and its analogue M344, target leukotriene A4 hydrolase (LTA4H), a key enzyme in LTB4 biosynthesis, to inhibit LTB4 biosynthesis. This reduces neutrophil migration and attenuates sepsis-associated ALI [202]. Furthermore, HDACis-mediated increases in histone acetylation levels inhibited NET formation (NETosis) and converted neutrophil death to apoptosis. Interestingly, in the presence of NETosis-promoting stimuli, high levels of HDACis limited NETosis and apoptosis and promoted neutrophil survival [203]. This suggests that HDACs can promote neutrophil death, leading to an explosive release of NETosis and inflammatory factors that exacerbate inflammation.

There are few studies on the regulation of neutrophils by HDACs in ALI, but there are similar studies in other organ inflammatory diseases. Butyrate, an HDAC inhibitor, modulates leukocyte activation, innate immune response, and oxidative stress to inhibit neutrophil migration capacity, formation of NETs, and secretion of pro-inflammatory cytokines in inflammatory bowel disease (IBD), thereby improving IBD [204]. In addition, aging increases susceptibility to alcoholic liver injury in mice and humans by downregulating the neutrophil SIRT1-C/EBPα-miR-223 axis [205]. The transcription factor forkhead box protein O3a (FOXO3a) regulates the expression of pro-angiogenic factors in neutrophils, and its activity is controlled by PTM such as acetylation and phosphorylation. Deacetylation of FOXO3a mediated by Sirt1 allows FOXO3a to remain in the nucleus and is further activated, while the deletion of Sirt1 allows increased ERK and AKT activity to promote FOXO3a phosphorylation, leading to FOXO3a translocation to the cytoplasm and inactivation [206]. FOXO3a is a key transcription factor controlling neutrophil angiogenic switch and sirt1 mediates its activation and promotes pro-angiogenic activity in interferon-deficient tumor neutrophils [206]. Dunnione, a powerful substrate for NADPH quinone oxidoreductase 1 (NQO1), restores NAD^+^-dependent histone deacetylase Sirt1 activity by increasing intracellular NAD^+^ levels. Sirt1 reduces the ability of neutrophils to produce NETs and reduces cancer thrombus formation by promoting histone deacetylation [207].

Neutrophils are functionally complex, and both the classical HDAC family, and Sirtuins have been found to play important roles in neutrophils. However, there are still some HDAC members with unproven specific roles in neutrophils, but each named HDAC is indispensable, and it is believed that the potential roles of these HDACs can be discovered in the future.

## 5. Targeting HDACs

We already know that mammalian HDACs include the classical HDAC family (class I, II, and IV HDACs) and the Sir2 family (class III HDACs), and although both belong to the HDACs and can exert deacetylation, the two families play very different roles in cellular processes. Although we have identified different functions of HDACs, such as regulation of transcription, regulation of non-histone substrates, and regulation of other PTMs, we would prefer to find natural products or synthetic compounds that target HDACs, so that they can be applied to clinical therapies depending on the need. To this end, a great deal of exploration has been conducted to discover the substances that activate HDACs and inhibit HDACs (Table 2). Not only does this represent a major advance in medicine, but it also facilitates further research on HDACs. 

### 5.1. Targeting Classical HDAC Family

The classical HDAC family regulates a variety of cellular processes, such as cell division, proliferation, gene transcription, and microtubule stability. As described earlier, the classical HDAC family can amplify inflammation, enhance EMT processes, and increase NETosis formation in ALI, with only a small fraction of HDACs playing a protective role. In addition to playing an unpopular role in ALI, the same is true in cancer and diabetes. Thus, natural products or synthetic compounds that inhibit the classical HDAC family have become substances of interest. 

Nature, the greatest pharmacologist, can produce a wide range of natural products that target histone deacetylases. The ability of n-Butyrate to increase histone acetylation levels in Hela and Friend erythroleukemia cells were discovered as early as the 1970s [208]. A subsequent study confirmed that n-Butyrate increases histone acetylation levels by inhibiting the deacetylation process [209], opening an era of exploration for targeting classical HDAC family compounds. Butyrate is a carboxylic acid HDACi with low potency and a short half-life in the human body, and there are no examples of such compounds that have been approved by the FDA for the time being. Trichostatin A (TSA) is a Streptomyces product that induces Friend cell differentiation. In 1990, TSA was found to prolong the half-life of acetylation on histones and was noncompetitively inhibited by TSA in partially purified histone deacetylases from wild-type mouse mammary tumor cells FM3A. Importantly, the reduced sensitivity of histone deacetylase to TSA in mutant FM3A cells with TSA resistance provides evidence for TSA targeting of histone deacetylase [210]. TSA was the first natural product found to have strong histone deacetylase inhibitory activity. Trapoxin is a fungal cyclic peptide that, unlike TSA, has irreversible inhibition of histone deacetylase and binds strongly to histone deacetylase [211], HDAC1 was isolated and purified by this binding force [27]. FK228 (romidepsin) is a potent antitumor antibiotic [212], as HDACi was approved by the FDA in 2009.

Suberoylanilide hydroxamic acid (SAHA) was designed and synthesized as a compound that strongly induces erythrogenic differentiation and was found to inhibit the enzymatic activity of HDAC1 and HDAC3 and was the first HDACi approved for cancer chemotherapy [213,214]. Both SAHA and TSA belong to the hydroxamic acid class of HDAC inhibitors with a high potency of action and were subsequently found to be pan-HDACi targeting class I, II, and IV HDACs. CI-994 and MS-275 (Entinostat) are benzamide derivatives with histone deacetylase inhibitory effects [215], which have high inhibitory activity in cells [216].

Tubacin is a specific inhibitor of HDAC6 and effectively inhibits HDAC6, tubacin increases the acetylation level of microtubulin in cells without affecting histones, due to the specificity of HDAC6 substrates [217]. Benzamide inhibitors such as MS-275 inhibit HDAC1, HDAC2, and HDAC3. HDAC8 in class I HDACs is not included in the inhibition, which may be related to the low homology between HDAC8 and other class I enzymes. Furthermore, despite the low deacetylation activity of class IIa HDACs, it still plays an essential role in the deacetylation process. 2-trifluoroacetylthiophene derivatives were found to be a specific substrate for class IIa HDACs [218], thus, becoming a specific inhibitor of class IIa HDACs.

### 5.2. Targeting Sir2 Family

The Sir2 family, Sirtuins, includes class III HDACs that regulate metabolism, stress response, and the aging process. Sirtuins are an attractive target for which many explorations have been made, identifying Sirtuin activators and Sirtuin inhibitors and using them as tools to study Sirtuins in depth. The association of Sirtuins with aging has led to the use of these natural products or synthetic compounds to explore diseases associated with aging, such as neurodegenerative diseases, diabetes, etc.

As Sirtuin activators, both Piceatannol and Resveratrol target Sirt1, the most important member of Sirtuins, and are also able to affect Sirt3 and Sirt5 [219,220]. Resveratrol, found in grapes and red wine, is a natural product and is also the most potent of the described Sirtuins activators. 1,4-DHP derivative is another natural product that activates sirt1 in vivo and in vitro, and also affects Sirt2 and Sirt3 [221]. SRT1720 is a synthetic Sirt1 activator that was designed to be synthesized during the exploration of drugs for the treatment of type 2 diabetes [222]. SRT2104 is a synthetic, highly specific activator of Sirt1, and no serious adverse effects have been observed [223].

In 2001, a phenotypic screen revealed the Sirtuin inhibitor-Cambinol, which inhibited yeast Sir2p gene silencing activity in vivo and in vitro, and human Sirt2 activity in vitro [224]. Ex-527 was found to use a unique NAD+-dependent deacetylation mechanism to exert its inhibitory effect, targeting Sirt1 and Sirt3 [225]. AGK2, a Sirt2-selective inhibitor, was found to ameliorate α-synuclein-mediated toxicity in Parkinson’s models [226].

In addition, there are several Sirtuins inhibitors, such as Compound 28e [227], Compound 8 [228], ELT-11c [229], UBCS0137 (Compound 39) [230], Compound 15e [231], SirReal2 [232], 3′-(3-fluoro-phenethyloxy)-2-anilinobenzamide [233], etc. The mechanisms by which they exert their inhibitory effects have not yet been investigated; nevertheless, these compounds are still exciting.

The discovery of HDAC inhibitors, Sirtuin activators, and Sirtuin inhibitors is a great help for the study of HDACs, and it also implies the possibility of discovering more highly specific activators or inhibitors and using them as clinical drugs in the future. The ability of these compounds to target other targets along with HDACs prompts questions about the feasibility of bifunctional HDAC therapeutics, such as inhibitors of both class I HDACs and kinase [234].

## 6. Conclusions

The mechanisms of ALI/ARDS progression involve a variety of cell types, including macrophages, vascular endothelial cells, alveolar epithelial cells, and neutrophils. We found that HDACs are closely associated with these types of cells.

The 18 HDACs include the classical HDAC family and Sirtuin. In ALI macrophages, HDACs are associated with the activation of macrophage inflammatory pathways. In ALI VECs, HDACs affect the glycocalyx stability and contraction of endothelial cells. In ALI AECs, HDACs act on their epithelial-mesenchymal transition. In ALI neutrophils, HDACs act on the migration and activation of neutrophils.

In general, most of the classical HDAC families play a pro-inflammatory role, and Sirtuins can play a role in improving inflammation in ALI. However, the research seen so far is only the tip of the iceberg. For example, HDAC11 can inhibit the release of inflammatory mediators from neutrophils, and there are too many potential mechanisms of action yet to be discovered. The downside is that a large number of HDACs have not been studied with different cell types, and their role between different pathways is unknown. In addition, the role of each HDAC for specific genes, the linkage between HDACs, and other molecular chaperones remain to be investigated. We hope that in the future HDACs will be used more often in ALI treatment.

## Figures and Tables

**Table 1:** The 18 mammalian HDACs.

Family	Class	Homology (*S. cerevisiae*)	Isoform	Localization	Key Features
Classical HDAC family (histone deacetylase family)	Class I HDACs	RPD3, HOS	HDAC1	Nucleus	Exist in NuRD, Sin3A, CoREST, and MiDAC complexes
			HDAC2		
			HDAC3	Nucleus/Cytoplasm	Exist in NCoR/SMRT
			HDAC8		Long-chain acyl lysine substrates
	Class IIa HDACs	HDA1	HDAC4	Nucleus/ Cytoplasm	HDRP and MEF2-interacting transcriptional repressors
			HDAC5		
			HDAC7		
			HDAC9		
	Class IIb HDACs		HDAC6	Cytoplasm	CD1 and CD2 structural domains
			HDAC10		
	Class IV HDACs	——	HDAC11	Nucleus	Long-chain acyl lysine substrates
Sir2 family (Sirtuin)	Class III HDACs	Sir2	Sirt1	Nucleus/ Cytoplasm	NAD^+^-dependent, Related to ADP/ATP
			Sirt2		
			Sirt3	Nucleus/Mitochondria	
			Sirt4	Mitochondria	
			Sirt5		
			SIrt6	Nucleus	
			Sirt7		

**Table 2:** Natural products and synthetic compounds targeting HDACs.

Compound Name	Compound Characteristics	Compound Targets	References
*HDAC inhibitor*			
n-Butyrate	Carboxylic acid	Class I, Class IIa, and Class IV HDACs	[208,209]
Trichostatin A (TSA)	Hydroxamic acid, Streptomyces product	Class I, Class II, and Class IV HDACs	[210]
Trapoxin	Fungal cyclopeptide, irreversible inhibitor	Class I, Class IIa, and Class IV HDACs	[211]
FK228 (romidepsin)	Potent antitumor antibiotic, approved by FDA in 2009	Class I, Class IIa, and Class IV HDACs	[212]
Suberoylanilide hydroxamic acid (SAHA)	Hydroxamic acid, a synthetic compound for induction of erythrogenic differentiation, approved by the FDA in 2006	Class I, Class II, and Class IV HDACs	[213,214]
MS-275 (Entinostat)	Benzamide derivative	HDAC1, HDAC2, HDAC3	[215,216]
Tubacin	——	HDAC6	[217]
2-trifluoroacetylthiophene derivatives	Class IIa HDACs specific substrate	Class IIa HDACs	[218]
*Sirtuin activator*			
Piceatannol	Natural product	Sirt1, Sirt3, Sirt5	[219]
Resveratrol	Natural products in grapes and red wines	Sirt1, Sirt3, Sirt5	[220]
1,4-DHP derivative	Natural products	Sirt1, Sirt2, Sirt3	[221]
SRT1720	Synthesis, selective	Sirt1	[222]
SRT2104	Synthesis, selective	Sirt1	[223]
*Sirtuin inhibitor*			
Cambinol	Natural product	One of the first Sirtuin inhibitors discovered	[224]
Ex-527	Competitive inhibition of nicotinamide binding	Sirt1, Sirt2, Sirt3	[225]
AGK2	——	Sirt2	[226]
Compound 28e	Synthetic, selective	Sirt1, Sirt2, Sirt3	[227]
Compound 8	——	Sirt1, Sirt2, Sirt3	[228]
ELT-11c	——	Sirt1, Sirt2, Sirt3	[229]
UBCS0137 (Compound 39)	——	Sirt1, Sirt2, Sirt3, Sirt5	[230]
Compound 15e	——	Sirt1, Sirt2, Sirt3	[231]
SirReal2	——	Sirt1, Sirt2, Sirt3, Sirt4, Sirt5, Sirt6	[232]
3′-(3-fluoro-phenethyloxy)-2-anilinobenzamide	Synthetic, selective	Sirt1, Sirt2	[233]

## Data Availability

Not applicable.
